# Bluetongue Virus in the Iberian Lynx (*Lynx pardinus*), 2010–2022

**DOI:** 10.3201/eid3010.240235

**Published:** 2024-10

**Authors:** Javier Caballero-Gómez, Marta Sánchez-Sánchez, Cristina Lorca-Oró, Isabel G. Fernández de Mera, Irene Zorrilla, Guillermo López, Rosa Rosell, Rebeca Grande-Gómez, Juan I. Montoya-Oliver, Javier Salcedo, Jorge Paniagua, Cristina Cano-Gómez, Moisés Gonzálvez, Ignacio García-Bocanegra

**Affiliations:** University of Cordoba, Cordoba, Spain (J. Caballero-Gómez, J. Paniagua, M. Gonzálvez, I. García-Bocanegra);; Carlos III Health Institute, Madrid, Spain (J. Caballero-Gómez, I. García-Bocanegra);; Institute for Game and Wildlife Research, Ciudad Real, Spain (M. Sánchez-Sánchez, I.G. Fernández de Mera);; Campus de la Universitat Autònoma de Barcelona, Bellaterra, Spain (C. Lorca-Oró, R. Rosell);; Junta de Andalucía, Malaga, Spain (I. Zorrilla, G. López);; Generalitat de Catalunya, Barcelona, Spain (R. Rosell);; Technical Assistance from the General Direction of the Natural Environment and Sustainable Development of the Board of Communities of Castilla La Mancha, Toledo, Spain (R. Grande-Gómez);; Ministry for the Ecological Transition and the Demographic Challenge, Madrid (J.I. Montoya-Oliver);; Junta de Andalucía, Seville, Spain (J. Salcedo);; Spanish Ministry of Agriculture, Food and Fisheries, Madrid (C. Cano-Gómez);; University of Murcia, Murcia, Spain (M. Gonzálvez).

**Keywords:** Bluetongue virus, viruses, Epidemiology, Iberian lynx, *Lynx pardinus*, Iberian ecosystems, Surveillance, Spain

## Abstract

Clinical infection and death caused by bluetongue virus infection has been reported in the Eurasian lynx. Bluetongue virus surveillance in the Iberian lynx revealed widespread and repeated exposure to serotypes 1 and 4 in wild and captive populations of this species. This exposure is possibly from a spillover event from sympatric ruminants.

Bluetongue virus (BTV) is a reemerging, multihost orbivirus of animal health concern. Although the disease is subject to eradication in some regions of Europe, BTV continues to circulate and spread to new areas of the continent (*1*,*2*). Wild ruminants play a role in the continued presence and dissemination of BTV in Iberian ecosystems (*3*), where they share habitats and natural resources with other wildlife species such as the Iberian lynx (*Lynx pardinus*). The Iberian lynx, endemic to the Iberian Peninsula, is one of the most vulnerable felids in the world (*4*). In recent decades, shared pathogens with sympatric species, including ruminants, have caused clinical disease and death in the Iberian lynx (*5*). Although exposure, clinical infection, and death because of BTV infection have been reported in the Eurasian lynx (*L. lynx*) (*6*,*7*), the susceptibility of Iberian lynxes to BTV is unknown. The objectives of our research were to determine BTV exposure in Iberian lynx populations, to assess potential risk factors associated with viral exposure in this species, and to evaluate the dynamics of seropositivity to BTV in longitudinally sampled animals.

## The Study

**Figure 1:**
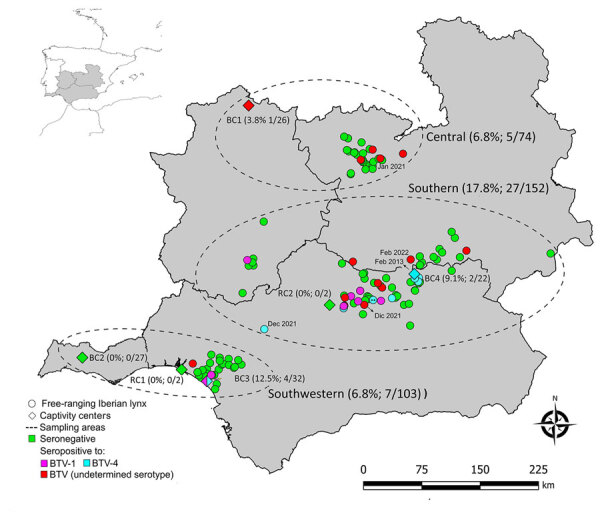
Spatial distribution and BTV serologic results of the Iberian lynxes (*Lynx pardinus*) sampled in the Iberian Peninsula during 2010–2022. The frequency of seropositivity and the numbers of seropositive and total of animals analyzed by ELISA at each sampling region and captivity center are shown in parentheses. Inset map shows location of study area on the Iberian Peninsula. **Animal tested positive for BTV RNA. BC, breeding center; BTV, bluetongue virus; RC, recovery center of threatened species.

We collected blood, serum, and spleen samples from 340 Iberian lynxes (229 free-ranging and 111 captive) throughout the Iberian Peninsula during 2010–2022 (Table; Figure 1). In addition, longitudinal surveillance was conducted on 50 of the 340 Iberian lynxes during the study period.

**Table:** Bluetongue virus seropositivity in Iberian lynxes (*Lynx pardinus*) sampled in the Iberian Peninsula during 2010–2022 by age, sex, life condition, sampling period, and region

Variable	Seroprevalence, % (95% CI)	No. positives/no. analyzed*	p value†
Age			
Yearling	4.5 (1.9–10.0)	5/112	<0.001
Subadult	6.0 (2.5–13.2)	5/83	
Adult	20.6 (14.7–28.0)	29/141	
Sex			
F	7.5 (4.3–12.6)	12/161	0.016
M	15.6 (10.1–21.8)	26/167	
Life condition			
Captive	6.3 (3.1–12.5)	7/111	0.025
Free ranging	14.0 (10.1–19.1)	32/229	
Sampling period‡			
2010–2016	12.4 (7.8–19.2)	16/129	0.128
2017–2020	7.3 (3.9–13.2)	9/124	
2021–2022	16.1 (9.8–25.2)	14/87	
Sampling region			
Central	6.8 (2.9–14.9)	5/74	0.009
South	17.8 (12.5–24.6)	27/152	
Southwest	6.8 (3.3–13.4)	7/103	

*Missing values excluded. †p<0.5 significant. ‡The frequency of seropositivity by sampling year was 16.7% (2/12) in 2010, 0% (0/1) in 2011, 11.1% (2/18) in 2012, 13.3% (2/15) in 2013, 0% (0/2) in 2014, 12.5% (3/24) in 2015, 12.3% (7/57) in 2016, 11.8% (4/34) in 2017, 13.3% (2/15) in 2018, 6.5% (2/31) in 2019, 2.3% (1/44) in 2020, 11.7% (9/77) in 2021, and 50.0% (5/10) in 2022.

We tested serum samples for BTV antibodies by using a double recognition ELISA (Gold Standard Diagnostics, https://www.goldstandarddiagnostics.com). Whenever possible, we tested ELISA-positive serum by using a virus neutralization test (VNT) to detect specific antibodies against BTV-1 and BTV-4 serotypes (Appendix). We assessed the presence of viral RNA in blood and spleen samples from ELISA-seropositive lynxes with available samples (n = 30) and a subset of randomly selected seronegative animals (n = 69) by molecular analyses (Appendix) (*8*–*10*). We analyzed associations between the seropositivity to BTV detected by ELISA and explanatory variables by using Pearson χ^2^ or Fisher exact tests, considering differences significant at p<0.05.

ELISA testing found that 39 (11.5%; 95% CI 8.5–15.3) of the 340 Iberian lynxes were positive for BTV antibodies (Table; Figure 1). We detected significant differences in the ages, sexes, life condition, and sampling region of the animals (Table). Of the 50 longitudinally sampled animals, 4 were seropositive at all samplings, whereas seroconversions were found in 9 animals and seroreversions in 3 animals (Appendix Table 1).

**Figure 2:**
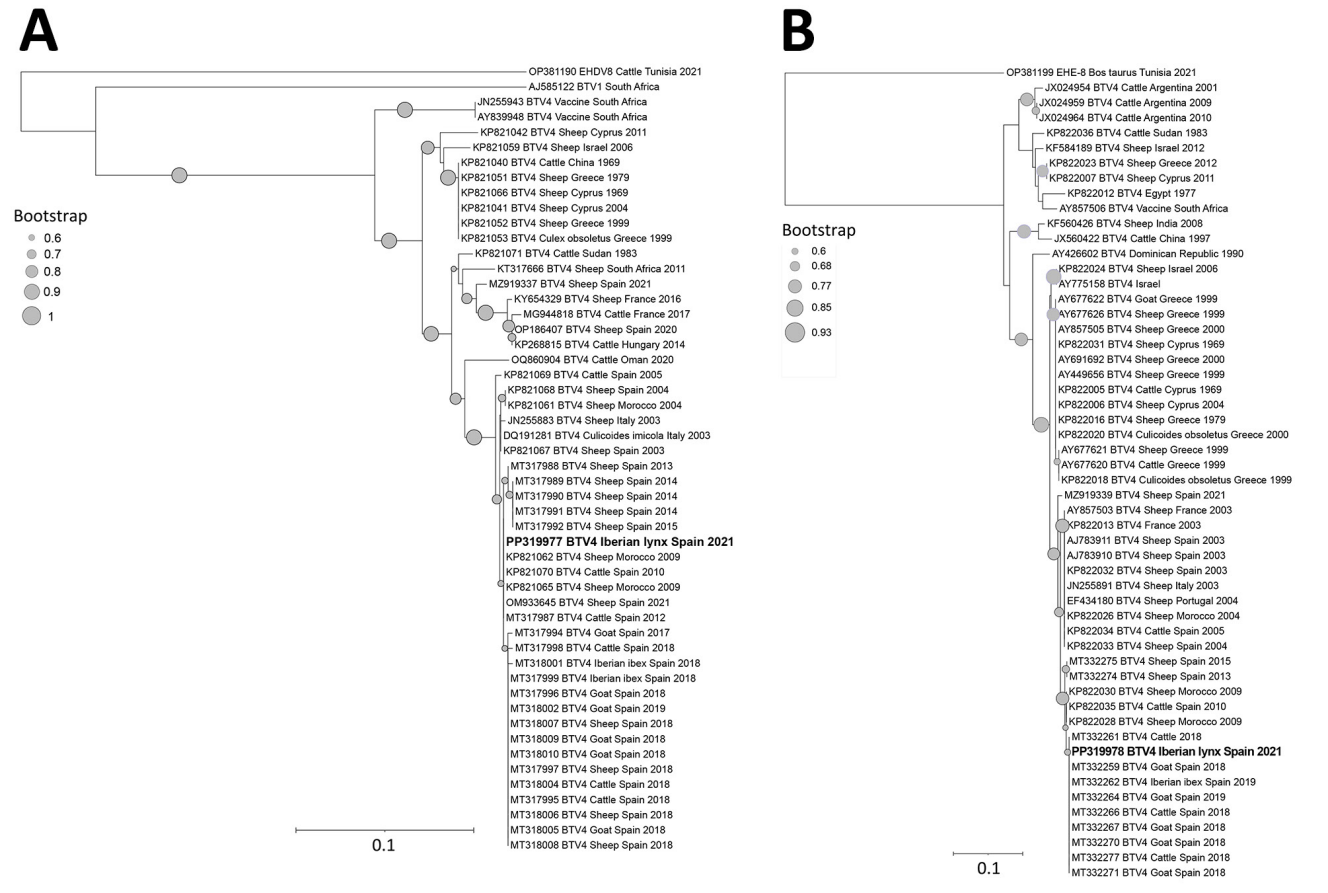
Phylogenetic tree of BTV from Iberian lynxes (*Lynx pardinus*) sampled in the Iberian Peninsula during 2010–2022 (bold) and reference sequences. Tree was constructed by the neighbor-joining method (1,000 bootstraps). A) Segment 2 tree, based on 393 nucleotides of 52 sequences. BTV-1 serotype and epizootic hemorrhagic disease virus serotype 8 reference strains were included to root the tree. B) Segment 10 tree, based on 216 nucleotides of 54 sequences. Epizootic hemorrhagic disease virus serotype 8 reference strain was included to root the tree. Gray circles indicate bootstrap values; only values ≥60 are shown. GenBank accession numbers are provided. Scale bars indicate number of substitutions per site. BTV, bluetongue virus.

Of the ELISA-seropositive samples, 7 could not be analyzed by using VNT because of cytotoxicity or insufficient sample volume, and 13 of the ELISA-seropositive samples were negative by using VNT (Appendix Table 2). Of the Iberian lynxes analyzed, 19 (59.4%) had antibodies against BTV by both ELISA and VNT. Specific antibodies against serotype BTV-1 were detected in 10 samples (3.0%, 95% CI 1.6%–5.4%) and against BTV-4 were detected in 9 samples (2.7%, 95% CI 1.4%–5.1%). Of note, 2/10 animals with BTV-1 antibodies also showed neutralizing activity to BTV-4. Despite having 4-fold lower titers, co-exposure to both serotypes in these animals cannot be ruled out. BTV RNA was detected in the blood of 1 Iberian lynx (1.0%, 95% CI 0.2%–5.5%), which also had specific neutralizing antibodies against BTV-4. Phylogenetic analysis of segments 2, 5, and 10 confirmed the presence of BTV-4 RNA (GenBank accession nos. PP319976–319978) and showed high similarity (>99%) with BTV-4 sequences obtained during the study period from cattle, goats, sheep, and Iberian ibexes in southern Spain (Figure 2).

Our study confirms the exposure of Iberian lynx populations to BTV, increasing the number of wildlife species susceptible to this virus. Although BTV was thought to be transmitted to carnivores through *Culicoides* biting midges, BTV infection in carnivores appears to occur primarily through the ingestion of infected animals (*11*). Because of this transmission, the higher seropositivity found in free-ranging animals (14.0%) compared with captive animals (6.3%) might be explained by the trophic behavior of free-ranging Iberian lynxes on wild ungulates (*12*), which is further supported by the high homology (>99%) of the BTV-4 sequence with those found in wild ungulates from Spain. However, it is also possible that free-ranging lynxes are more frequently exposed to *Culicoides* because of their larger home range and the lack of periodic cleaning and disinfestation procedures used in captivity centers (*13*), which could interfere with the breeding cycle of *Culicoides*.

The higher seroprevalence detected in male compared with female animals aligns with findings in wild ruminants, where male animals’ larger home ranges and body mass increase their risk for contact with *Culicoides* midges (*14*). The higher frequency of seropositivity detected in adults (20.6%) compared with that for subadults (6.0%) and yearlings (4.5%) might be associated with a higher likelihood of cumulative exposure to BTV and lifelong persistence of antibodies in Iberian lynxes (*3*). Of note, 1 yearling sampled in 2013, 3 yearlings sampled in 2021, and 1 yearling sampled in 2022 were positive for BTV antibodies, suggesting recent exposure to BTV in Iberian lynx populations during these years. This hypothesis is supported by concurrent outbreaks of BTV infection in livestock (*15*). Furthermore, the 4 BTV-1 seroconversions overlap spatiotemporally with outbreaks of this serotype reported in livestock (*15*). This finding, together with the higher seroprevalence observed in lynxes from the southern region, which is consistent with the higher number of BTV outbreaks reported in livestock in this area (*15*), suggests a common epidemiologic pattern of BTV in domestic and wild species. However, 2/5 seropositive yearlings were sampled in the provinces of Jaen and Toledo (southern and central Spain) in February 2013 and January 2021, whereas the previous BTV outbreaks reported in livestock in these provinces occurred in 2010 and 2014 (*15*). Those findings are consistent with previous reports of BTV circulating in wild ruminants in the absence of BTV outbreaks in livestock in Spain (*3*) and highlight the importance of wildlife in the epidemiology of this virus in the study area, especially in areas where livestock vaccination has been implemented.

## Conclusions

We confirmed the presence of BTV-1 and BTV-4 serotype neutralizing antibodies in both free-ranging and captive Iberian lynxes. This result is consistent with the BTV outbreaks detected in livestock in Spain during the study period, where cases of BTV-1 and BTV-4 were reported (*15*). The detection of BTV-4 RNA in an Iberian lynx confirms the susceptibility of the species to BTV infection. The infected lynx was a BTV-4–seropositive, free-ranging lynx sampled in southern Spain (municipality of Marmolejo) on November 31, 2021. Exposure to BTV was also detected in a yearling sampled close to this municipality on December 3, 2021, and outbreaks of BTV-4 serotype were reported in domestic ruminants close to this location during this period (*15*). This finding, together with the high similarity with other sequences obtained from domestic and wild ruminants in Spain, provides evidence that the exposure of Iberian lynxes to BTV could be the result of spillover from domestic or wild sympatric ruminants, indicating a common epidemiologic cycle of BTV among ruminants and Iberian lynxes.

In summary, we report evidence of widespread and repeated exposure to BTV in Iberian lynx populations. Further studies are needed to determine the effects of BTV on the health of Iberian lynx populations, but the low prevalence of infection suggests that this species may be considered a spillover host, rather than a true reservoir of BTV. 

Appendix Additional information for study of bluetongue virus in the Iberian lynx (*Lynx pardinus*), 2010–2022.
