# Supplementary material for: Bluetongue Virus in the Iberian Lynx (Lynx pardinus), 2010–2022
Source: Emerg Infect Dis. 2024 Oct;30(10):2169–73. doi: 10.3201/eid3010.240235 (PMC11431934; doi:10.3201/eid3010.240235)
Supplement: Appendix — Additional information for study of bluetongue virus in the Iberian lynx (Lynx pardinus), 2010–2022. [file 24-0235-Techapp-s1.pdf]

Article DOI: https://doi.org/10.3201/eid3010.240235

EID cannot ensure accessibility for supplementary materials supplied by authors. Readers who have difficulty accessing supplementary content should contact the authors for assistance.

Bluetongue Virus in the Iberian Lynx (*Lynx pardinus*), 2010–2022

Appendix

Sampling

We collected blood, spleen and/or serum samples from 340 Iberian lynxes throughout the Iberian Peninsula during the period 2010–2022 (Table 1; Figure 1). Of these, a total of 229 were free-ranging animals from the three areas where this species is mainly distributed (central, southern and southwestern free-range area) and 111 were lynxes kept in captivity in the four captive breeding centers (BC1-BC4) and in three recovery centers of threatened species (RC1-RC3) of the Iberian lynx ex situ conservation program. We also performed longitudinal surveillance on 50 of the 340 Iberian lynxes sampled during the study period.

Blood and/or spleen samples, including those from the longitudinal survey, were collected from individuals subjected to health programs, medical check-ups, or necropsy at the Wildlife Recovery Center “El Chaparrillo” (Castilla-La Mancha, central Spain) and the Center for Analysis and Diagnosis of Wildlife (CAD, Andalusia, southern Spain) during 2010–2022. Alive animals were chemically immobilized with a combination of ketamine (2.5 mg/kg, Imalgene 1000®, Merial, Barcelona, Spain), dexmedetomidine (0.02 mg/kg; Sedadex®, Dechra, Barcelona, Spain) and midazolam (0.4 mg/kg; Normon®, Madrid, Spain) administered intramuscularly. Blood samples were collected into sterile tubes with EDTA and without anticoagulant were obtained from each animal by venipuncture of cephalic or jugular vein in alive animals and form heart in deceased individuals. Sera from blood of tubes without anticoagulant were obtained after centrifugation at 400 × g for 15 min. Blood and sera were stored at −80°C until laboratory analysis.

This study did not involve the intentional killing of animals. Iberian lynxes were sampled by authorized veterinarians and animal keepers following routine procedures on alive and dead

individuals before the design of this study (*1*), in compliance with Ethical Principles in Animal Research. Whenever possible, epidemiologic information from each individual was recorded, including sex, age (yearlings: <1 year old; subadults: 1 to 3 years old; adults: >3 years old), life condition (free-ranging versus captivity), sampling date and georeferenced location.

Serologic analyses

Whenever possible, ELISA-positive sera were tested for the detection of specific antibodies against BTV-1 and BTV-4 (the serotypes circulating in the study area during the study period) by virus neutralization test (VNT), as previously described (*2*). Briefly, serum samples were inactivated at 56°C for 30 min and serially diluted (1:4–1:512) in MEM (Eagle's minimum essential medium). Sera were then mixed with 100 TCID50 (50% tissue culture infective doses) of each reference strain of BTV-1 (BTV-1/ALG/2006) and BTV-4 (BTV-4/SPA/2004) and incubated in 96-well plates at 37°C for 1 h 30 min. Finally, 100 µl of Vero E6 cell suspension (1.5×10^4 cells/well) in cell growth media (MEM supplemented with 5% fetal calf serum, 300 µg L-glutamine/ml, 300 U penicillin/ml and 300 µg streptomycin/ml) was added to each well. The mixture was further incubated at 37°C for 6–7 days until a cytopathic effect (CPE) developed in control wells containing 100 $TCID_{50}$ of virus and no serum. Samples were considered positive only if they showed neutralization (absence of CPE) at dilutions ≥1:4 (*3*). The neutralizing immune response observed was considered specific when VNT titers for a given serotype were ≥4-fold higher than titers obtained for the other serotype (*2*). Samples showing ≤2-fold differences between VNT titers were considered positive but inconclusive for serotype and were, therefore, excluded to calculate seroprevalence by serotype.

Molecular analyses

Whenever possible, seropositive animals and a subset of random selection of seronegative animals were subjected to molecular analyses. RNA from blood (n = 99) and/or spleen (n = 14) samples from 99 individuals were extracted using using Tri Reagent® (Sigma-Aldrich, Burlington, MA, USA), according to the manufacturer’s instructions. RNA concentration, quality and purity were checked using a NanoDrop One® spectrophotometer (ThermoScientific, Waltham, MA, USA) and then stored at −80°C. RNA samples were screened

by a real-time RT-PCR targeting a 75 bp fragment of segment 5 of BTV. Primers and probe were BTV-S5-F (5′-GGCAACYACCAAACATGGA-3′), BTV-S5-R (5′-AAAGTYCTCGTGGCATTWGC-3′), and BTV-S5-P (5′-FAM-CYCCACTGATRTTGTATTTTCTCAA-TAMRA-3′), as previously described (*4*). All reactions were performed on the CFX96 Touch real-time PCR Detection System (Bio-Rad, Hercules, CA, USA), using the SuperScript III Platinum One-Step qRT-PCR Kit (ThermoFisher, MA, USA), according to manufacturer's protocol. Positive samples were shipped to Spanish National Reference Laboratory for Bluetongue (LCV, Algete, Madrid) for confirmation and serotyping by real-time RT-PCR and sequencing. In this case, an automated RNA extraction was performed using magnetic beads technology, and both real-time RT-PCR was carried out by AgPath-ID One-Step RT-PCR Reagents (Applied Biosystems, Foster City, CA, USA), according to the manufacture's protocol. A generic real-time RT-PCR targeting segment 10 (*5*) was carried out to confirm the presence of BTV RNA and BTV-1 and BTV-4-specific real-time RT-PCRs were subsequently conducted (*6*,*7*).

Phylogenetic analyses were performed on segments 2, 5 and 10 using three different endpoint RT-PCR, as previously described (*7*). For these assays, the GoTaq® G2 DNA Polymerase Master Mix (Promega) and MultiScribe Reverse transcription (Applied Biosystems) were used. The amplicons of these RT-PCRs were examined on 1.5% agarose gels stained with RedSafe Nucleic Acid Staining solution (iNtRON Biotechnology, Seongnam, Korea). Positive PCR products were purified with the QIAquick PCR Purification Kit (QIAGEN, Hilden, Germany). Sequencing reactions were performaed using the BigDye® Terminator v3.1 Cycle Sequencing Kit (Applied Biosystems) and analyzed with a 3130XL Genetic Analyzer (Applied Biosystems). The consensus sequences were obtained using SeqMan Software NGen® Version 12.0 (DNASTAR.Madison, WI, USA) and compared with published BTV sequences using online BLAST algorithm (https://blast.ncbi.nlm.nih.gov/Blast.cgi). Each isolate obtained in the present study was also compared with published BTV sequences using online BLAST analysis (https://blast.ncbi.nlm.nih.gov/Blast.cgi). ClustalW was used for nucleotide sequence alignments, with representative complete genome sequences of Seg-2 and Seg-10, of BTV-4 available from GenBank. The phylogenetic tree was reconstructed using the neighbor-joining method and a 1,000 bootstrap procedure. The final tree was obtained with MEGA 7 and edited in iTOLv6 online service (https://itol.embl.de/).

Appendix Table 1. Antibodies against bluetongue virus in longitudinally sampled Iberian lynxes. Black dots indicate presence of antibodies to bluetongue virus and white dots indicate absence of antibodies to bluetongue virus. When two samplings were carried out in the same year, the number of interval months is indicated as superscript.

Life condition	Origin	Interpretation	2010	2011	2012	2013	2015	2016	2017	2018	2019	2020	2021	2022
Free-ranging	S	Seroconversion	○	NA	NA	NA	●	NA	NA	NA	NA	NA	NA	NA
Free-ranging	SW	Negative at all samplings	NA	NA	○	NA	NA	○	NA	NA	NA	NA	○	NA
Free-ranging	SW	Negative at all samplings	NA	NA	NA	NA	NA	NA	NA	NA	NA	○	○	NA
Free-ranging	SW	Negative at all samplings	NA	NA	NA	NA	NA	○	○	○	NA	○	NA	NA
Free-ranging	SW	Negative at all samplings	NA	○	NA	NA	○	NA	NA	NA	NA	NA	NA	NA
Free-ranging	S	Positive at all samplings	NA	NA	NA	NA	●	NA	NA	●	NA	NA	NA	NA
Free-ranging	S	Negative at all samplings	NA	NA	NA	NA	NA	○	NA	NA	NA	NA	○	NA
Free-ranging	S	Seroconversion	NA	NA	NA	NA	○	NA	●*	NA	NA	NA	NA	NA
Free-ranging	BC4/S	Positive at all samplings	NA	NA	NA	NA	NA	NA	NA	NA	NA	●[†];●[5]	NA	NA
Free-ranging	S	Positive at all samplings	NA	NA	NA	NA	NA	NA	NA	NA	●*	●*	NA	NA
Free-ranging	S	Negative at all samplings	NA	NA	NA	NA	○	○	NA	NA	NA	NA	NA	NA
Free-ranging	NA/C	Negative at all samplings	NA	NA	NA	NA	NA	NA	NA	NA	○	NA	NA	○
Free-ranging	C	Negative at all samplings	NA	NA	NA	NA	NA	NA	NA	○	NA	NA	○	NA
Free-ranging	C/S	Negative at all samplings	NA	NA	NA	NA	NA	NA	NA	○	NA	NA	○	NA
Free-ranging	S	Seroconversion	NA	NA	NA	NA	NA	NA	NA	NA	NA	○	●	NA
Free-ranging	S/SW	Negative at all samplings	NA	NA	NA	NA	○	○	NA	NA	NA	NA	NA	NA
Free-ranging	C	Negative at all samplings	NA	NA	NA	NA	NA	NA	NA	○	NA	○	NA	NA
Free-ranging	S	Seroconversion	NA	NA	NA	NA	NA	○	NA	NA	NA	NA	●*	NA
Free-ranging	NA/C	Negative at all samplings	NA	NA	NA	NA	NA	NA	NA	NA	○	NA	○	NA
Free-ranging	S	Negative at all samplings	NA	NA	NA	NA	NA	NA	NA	○	NA	○	NA	NA
Free-ranging	S	Negative at all samplings	NA	NA	NA	NA	NA	○	NA	NA	NA	○	NA	NA
Free-ranging/ Captivity	S/BC4/ SW/RC2	Seroreversion and seroconversion	NA	NA	NA	●	NA	NA	NA	○	●*	NA	○	NA
NA/ Free-ranging	SW	Negative at all samplings	○	NA	○	NA	NA	NA	NA	NA	NA	NA	NA	NA
Captivity/ Free-ranging	BC4/S	Seroconversion	NA	NA	○	NA	●	NA	NA	NA	NA	NA	NA	NA
Captivity/ Free-ranging	BC2/S	Negative at all samplings	NA	NA	○	NA	NA	NA	NA	NA	NA	NA	NA	NA
Captivity/ Free-ranging	BC2/S	Negative at all samplings	NA	NA	○	NA	○	NA	NA	NA	NA	NA	NA	NA
Captivity/ Free-ranging	BC2/S	Seroconversion	NA	NA	NA	○	NA	NA	●*	NA	NA	NA	NA	NA
Captivity/ Free-ranging	S/C	Negative at all samplings	NA	NA	NA	NA	○	NA	NA	○	NA	NA	NA	NA
Captivity/ Free-ranging	BC1/S	Negative at all samplings	NA	NA	NA	NA	NA	○	NA	○	NA	NA	NA	NA
Captivity /Free-ranging	BC3/S	Negative at all samplings	NA	NA	NA	NA	NA	○	NA	NA	○	NA	NA	NA
Captivity	BC3	Negative at all samplings	NA	NA	○	NA	NA	○	NA	NA	NA	NA	NA	NA
Captivity	BC3	Negative at all samplings	NA	NA	○	NA	NA	NA	○	NA	NA	NA	NA	NA
Captivity	CC2	Negative at all samplings	○	NA	NA	NA	○	NA	NA	NA	NA	NA	NA	NA
Captivity	BC4	Negative at all samplings	NA	NA	○	NA	○	NA	NA	NA	NA	NA	NA	NA
Captivity	BC3	Negative at all samplings	NA	NA	○	NA	NA	○	NA	NA	NA	NA	NA	NA
Captivity	BC3/ BC4	Negative at all samplings	NA	NA	○	NA	NA	○	NA	NA	NA	NA	NA	NA
Captivity	BC3	Negative at all samplings	NA	NA	NA	NA	○	NA	○	NA	NA	NA	NA	NA
Captivity	BC2	Negative at all samplings	○	NA	○	NA	NA	NA	NA	NA	NA	NA	NA	NA
Captivity	BC3/ BC1	Negative at all samplings	○	NA	○	NA	NA	○	NA	NA	○	NA	NA	NA
Captivity	BC3	Seroreversion	NA	NA	●	NA	○	NA	NA	NA	NA	NA	NA	NA
Captivity	BC3	Seroreversion	NA	NA	NA	NA	NA	●	○; ○[1]	NA	NA	NA	NA	NA
Captivity	BC1/ BC4	Negative at all samplings	NA	NA	NA	NA	NA	○	○	NA	NA	NA	NA	NA
Captivity	BC3	Negative at all samplings	○	NA	NA	NA	NA	○	NA	NA	NA	NA	NA	NA
Captivity	BC1	Negative at all samplings	NA	NA	○	NA	NA	○	NA	NA	NA	NA	NA	NA
Captivity	RC3/ BC3	Negative at all samplings	NA	NA	○	○	○	NA	NA	NA	NA	NA	NA	NA
Captivity	BC3/ BC1	Seroconversion	NA	NA	○	●	NA	●	NA	NA	NA	NA	NA	NA
Captivity	BC1	Seroconversion	NA	NA	NA	NA	○	NA	○	NA	NA	NA	●	NA
Captivity	BC3/ BC4	Negative at all samplings	NA	NA	○	NA	NA	○	NA	NA	NA	NA	NA	NA

Life condition	Origin	Interpretation	2010	2011	2012	2013	2015	2016	2017	2018	2019	2020	2021	2022
Captivity	BC3/ RC1	Negative at all samplings	NA	NA	NA	NA	○	○	NA	NA	NA	NA	NA	NA
Captivity	BC4	Positive at all samplings	●	NA	NA	NA	●†	NA	NA	NA	NA	NA	NA	NA
**Total positives /Total analyzed**			**1/6**	**0/1**	**1/17**	**2/4**	**4/17**	**2/20**	**2/9**	**1/9**	**2/6**	**3/9**	**3/10**	**0/1**

BC, breeding center; C, central; NA, unknown; RC, recovery center of threatened species; S, south; SW, southwest.
*Neutralizing antibodies against BTV-1.
†Neutralizing antibodies against BTV-4

Appendix Table 2. Results of VNT for the detection of antibodies against bluetongue virus in seropositive Iberian lynx.

Age	Life condition	Sampling area	Sampling year	VNT titers* BTV-1	VNT titers* BTV-4	Interpretation
Adult	BC3	SW	2016	Negative	32	BTV-4
Adult	Free ranging	SW	2015	Negative	Negative	Undetermined BTV serotype
Adult	Free ranging	SW	2016	64	16	BTV-1
Adult	Free ranging	SW	2016	Negative	Negative	Undetermined BTV serotype
Adult	Free ranging	S	2017	64	8	BTV-1
Adult	Free ranging	S	2012	64	Negative	BTV-1
Adult	Free ranging	S	2021	Negative	4	BTV-4†
Adult	BC3	SW	2012	Negative	Negative	Undetermined BTV serotype
Adult	Free ranging	S	2018	Negative	16	BTV-4
Adult	Free ranging	S	2015	Negative	Negative	Undetermined BTV serotype
Adult	BC3	SW	2016	32	Negative	BTV-1
Adult	Free ranging	S	2015	Negative	Negative	Undetermined BTV serotype
Adult	Free ranging	S	2016	Negative	8	BTV-4
Adult	Free ranging	S	2019	128	Negative	BTV-1
Yearling	BC4	S	2013	Negative	Negative	Undetermined BTV serotype
Adult	Free ranging	S	2016	Negative	16	BTV-4
Adult	BC1	S	2021	Negative	Negative	Undetermined BTV serotype
Adult	Free ranging	S	2017	32	Negative	BTV-1
Adult	Free ranging	S	2017	Negative	12	BTV-4
Adult	Free ranging	S	2017	64	Negative	BTV-1
Adult	Free ranging	S	2020	Negative	8	BTV-4
Adult	Free ranging	S	2019	64	Negative	BTV-1
Adult	Free ranging	S	2018	32	Negative	BTV-1
Adult	Free ranging	S	2021	Negative	Negative	Undetermined BTV serotype
Adult	Free ranging	S	2021	64	Negative	BTV-1
Adult	Free ranging	S	2021	Negative	4	BTV-4
Subadult	Free ranging	C	2022	Negative	Negative	Undetermined BTV serotype
Yearling	Free ranging	S	2021	Negative	Negative	Undetermined BTV serotype
Subadult	Free ranging	S	2022	Negative	Negative	Undetermined BTV serotype
Subadult	Free ranging	S	2022	Negative	Negative	Undetermined BTV serotype
Yearling	Free ranging	S	2021	Negative	8	BTV-4
Subadult	Free ranging	C	2022	Negative	Negative	Undetermined BTV serotype

BC, breeding center; C, central; S, south; SW, southwest; VNT, virus neutralization test.
*The titration of neutralizing antibodies against BTV could not be carried out in duplicate so the interpretation of titers should be conducted with caution
†This animal was also positive to BTV-4 RNA by RT-qPCR and sequencing

References

1. Rivas A, Boixader J, Vargas A, Pérez MJ, Serra R, Asensio V, et al. Manual del manejo del lince ibérico en cautividad. Programa de conservación ex-situ del Lince Ibérico. 2016 [cited 2024 Feb 11] https://www.lynxexsitu.es/ficheros/documentos_pdf/84/Manual_Manejo_Lince_Iberico_2016.pdf

2. World Organization for Animal Health. WOAH Terrestrial Manual. Bluetongue. 2021 [cited 2024 Jan 22]. https://www.oie.int/fileadmin/Home/eng/Health_standards/tahm/3.01.03_BLUETONGUE.pdf.

3. Lorca-Oró C, López-Olvera JR, Fernández-Sirera L, Solanes D, Navarro N, García-Bocanegra I, et al. Evaluation of the efficacy of commercial vaccines against bluetongue virus serotypes 1 and 8 in experimentally infected red deer (*Cervus elaphus*). Vet Microbiol. 2012;154:240–6. PubMed https://doi.org/10.1016/j.vetmic.2011.07.008

4. Toussaint JF, Sailleau C, Breard E, Zientara S, De Clercq K. Bluetongue virus detection by two real-time RT-qPCRs targeting two different genomic segments. J Virol Methods. 2007;140:115–23. PubMed https://doi.org/10.1016/j.jviromet.2006.11.007

5. Hofmann MA, Renzullo S, Mader M, Chaignat V, Worwa G, Thuer B. Genetic characterization of toggenburg orbivirus, a new bluetongue virus, from goats, Switzerland. Emerg Infect Dis. 2008;14:1855–61. PubMed https://doi.org/10.3201/eid1412.080818

6. Gómez-Guillamón F, Caballero-Gómez J, Agüero M, Camacho-Sillero L, Risalde MA, Zorrilla I, et al. Reemergence of bluetongue virus serotype 4 in Iberian ibex (*Capra pyrenaica*) and sympatric livestock in Spain, 2018–2019. Transbound Emerg Dis. 2021;68:458–66. PubMed https://doi.org/10.1111/tbed.13696

7. Maan S, Maan NS, Belaganahalli MN, Potgieter AC, Kumar V, Batra K, et al. Development and evaluation of real time RT-PCR assays for detection and typing of bluetongue virus. PLoS One. 2016;11:e0163014. PubMed https://doi.org/10.1371/journal.pone.0163014
